# Red chili powder dietary supplementation regularized the performance, hematobiochemical indices, oxidative status, and 8-hydroxy-2’-deoxyguanosine of aflatoxin B1 exposed broiler chickens

**DOI:** 10.1093/tas/txae006

**Published:** 2024-01-11

**Authors:** Olugbenga David Oloruntola

**Affiliations:** Department of Animal Science, Adekunle Ajasin University, Akungba Akoko, Nigeria

**Keywords:** DNA degeneration, growth metrics, mycotoxin, oxidative stress, phytogenic supplement, poultry

## Abstract

The effects of red chili powder dietary supplementation on the performance, hematobiochemical indices, oxidative status, and DNA damage in broiler chickens fed aflatoxin B_1_ (AFB_1_) contaminated diets were studied. Two hundred and forty Cobb 500 breed day-old broiler chicks were randomly distributed into control group (CONT), 0.5 mg/kg AFB1-exposed group (AFTB), 0.5 g/kg red chili pericarp powder supplementation alongside the 0.5 mg/kg AFB1 exposed group (RCPA), and 0.5 g/kg red chili seed powder supplementation alongside the 0.5 mg/kg AFB_1_ exposed group (RCSA). The red chili supplementation, in both pericarp powder and seed powder, positively influenced broiler performance by improving (*P* < 0.05) weight gain, feed intake, and feed conversion ratio, with a reduction in mortality rates compared to the AFTB group. Hematological indices showed that AFB1 exposure decreased (*P* < 0.05) the red blood cell count, packed cell volume, and hemoglobin (Hb) concentration, but the red chili supplementation mitigated these reductions. Additionally, total white blood cell counts were maintained (*P* > 0.05) in red chili-supplemented groups compared to CONT. Red chili supplementation increased (*P* < 0.05) the total protein and globulin concentrations and reduced (*P* < 0.05) liver enzyme levels compared to the AFTB group. The oxidative enzyme levels in RCPA and RCSA were similar (*P* > 0.05) to CONT groups. The red chili supplementations counteracted DNA damage, as reflected by similar (*P* > 0.05) 8-hydroxy-2’-deoxyguanosine levels recorded in RCPA, RCSA, and CONT groups levels. These findings suggest that 0.5 g/kg red chili supplementation has the potential to ameliorate the adverse effects of AFB_1_ exposure on broiler chickens, improving their performance and health.

## Teaser

Unlock the power of red chili! Discover how dietary supplementation with red chili pericarp and seed powder transforms the health and performance of broiler chickens exposed to aflatoxin B_1_ . From weight gain to oxidative balance, the findings reveal a spicy secret to boosting poultry resilience. Dive into the study and spice up your understanding of the impact of red chili on poultry well-being!

## Introduction

Poultry is a major animal industries with the quickest rate of growth and poultry meat is one of the most popular animal food sources consumed globally. In the tropics and subtropics, broiler chickens’ growth and health conditions are negatively affected by variables such as heat stress and nutritional factors such as aflatoxin B_1_ contamination of feed (Yunus et al., 2011; Mishra and Jha, 2019).

A diverse array of tropical and subtropical food and feed products and ingredients often carry aflatoxins, which are secondary byproducts of various Aspergillus species. Aflatoxins being hepatoxic and carcinogenic form the basis for their significant relevance in public health (Benkerroum, 2020a). The aflatoxins pervading an assortment of oilseed meals, dried fruits, spices, and cereals, have amplified the concern surrounding their contamination (van Egmond and Jonker, 2004; Yunus et al., 2011). AFB_1_’s toxicity, pathogenicity, oncogenicity, prevalence, and the difficulties associated with its control of the other hand make it a prominent concern in food safety and public health efforts and on the other hand makes it stand out as the most commonly encountered type of aflatoxin that carry greater detrimental effects compared to other strains of aflatoxins (Yunus et al., 2011). Notably, during the metabolic processes involving aflatoxin B_1_ (AFB_1_), there is formation of free radicals such as reactive oxygen species (ROS), which gives rise to oxidative damage (Yilmaz et al., 2017). These ROS contribute to tissue impairment through DNA oxidation, lipid peroxidation, protein oxidation, and depletion of thiol compounds. Oxidative stress is among the potential mechanisms underpinning AFB_1_-triggered cellular damage and DNA injury, culminating in oncogenesis in animals (Yilmaz et al., 2017). However, antioxidants exhibit the capability to mitigate the effects of oxidative stress by directly scavenging ROS, and by curbing cell proliferation via protein phosphorylation (Lobo et al., 2010). A range of therapeutic plants and dietary phyto-supplements, including Red Chilies, onion, garlic, common wireweed, mustard, clove, cinnamon, curry leaf, and ginger, among others, which have been recognized for their nutraceutical and functional attributes as antioxidants (Lobo et al., 2010; Olarotimi et al., 2023a; b).

A well-established correlation has been reported to exist between oxidative stress and feed toxins in poultry production (Mishra and Jha, 2019). For instance, the aflatoxin B1 contamination in feed contributes to reduced feed intake, consequently leading to suboptimal growth, immunosuppression, hypoxia, and elevated mortality rates (Mujahid et al., 2007; Song and King, 2015; Mishra and Jha, 2019). Beyond their influence on serum chemistry, aflatoxin B1 and oxidative stress also induce DNA chain breakage and mutations, (Yunus et al., 2011; Yilmaz et al., 2017).

Previous investigations elucidated the innate antioxidant properties and other nutraceutical properties within red chili fruit (Oloruntola et al., 2023). However, it is noteworthy that the properties and potentials, of the distinct components of red chili fruits i.e., seeds, pericarp, and placenta, displays variance (Lahbib et al., 2017; Palma et al., 2020). Upon this premise, this study was conducted to delve into the effects of introducing red chili pericarp powder and red chili seed powder into the diet of aflatoxin B_1_ exposed broiler chickens, particularly in the context of their performance characteristics, hematological indices, serum chemistry, oxidative status, and a marker indicative of DNA damage.

## Materials and Methods

### Animal ethics, location of the study area, red chilli fruits, aflatoxin B1, and experimental diets

The broiler chicken care and management procedure was approved by the Animal Welfare and Ethical Committee of the Department of Animal Science, Adekunle Ajasin University, Akungba Akoko, Nigeria. The mycotoxin research was carried out at the Teaching and Research Farm of Adekunle Ajasin University, Akungba Akoko, Nigeria. Fresh Red chilli fruits were procured from local farmers in Akure, Nigeria. The methods and procedures for processing red chilli fruits’ pericarp and seeds to their respective red chilli fruit pericarp powder and red chilli fruits’ seed powder (RSP), their analysis as well as the results, have been previously documented by Oloruntola et al. (2023). Briefly, the red chilli fruits were sliced with a clean stainless steel knife and subsequently, the seeds and pericarp of the fruits were separated, spread lightly on a stainless tray, and air-dried for a period of two weeks. Thereafter, the pericarp and seeds of the red chilli fruits were pulverized using a blender to create red chilli fruit pericarp powder and RSP, respectively.

Aflatoxin B_1_ (AFB_1_) was prepared by culturing *Aspergillus flavus* on maize grits and quantified for AFB_1_ (Mgbeahuruike et al., 2018). A broiler chickens diet (Table 1), was formulated and was divided into four equal portions. The first portion (CONT) was neither supplemented nor contaminated with AFB1, the second through the fourth portion (AFTB, RCPA, and RCSA) were contaminated with 0.5 mg/kg AFB1. In addition, the RCPA, and RCSA were supplemented with red chili pericarp powder and red chili, respectively. The experimental diets are defined as follows:

**Table 1.** Composition of the experimental diets

Ingredients, %	Broiler starter phase, 1 to 3 wk	Broiler finisher phase, 4 to 6 wk
Maize	50.36	58.36
Maize bran	3.00	0.00
Rice bran	0.00	3.02
Fish meal	3.00	3.00
Soybean meal	38.00	30.00
Bone meal	3.00	3.00
Premix	0.31	0.31
Limestone	0.49	0.47
Salt	0.31	0.31
Lysine	0.24	0.24
Methionine	0.29	0.29
Soy oil	1.00	1.00
Composition, %		
Metabolizable energy, Kcal/kg	3,018.1	3,108.2
Available phosphorus	0.48	0.43
Calcium	1.03	1.04
Crude fiber	3.52	3.58
Crude fat	4.23	2.38
Crude protein	22.17	20.04

CONT: Control. AFTB: 0.5 mg/kg AFB_1_ contamination. RCPB: 0.5 g/kg red chili pericarp powder supplementation and 0.5 mg/kg AFB_1_ contamination. RSAB: 0.5 g/kg red chili seeds powder supplementation and 0.5 mg/kg AFB_1_ contamination.

Procedures earlier reported by Olarotimi et al. (2023b) was used to prepare the experimental diets with the AFB_1_ contamination level of 0.5 mg/kg. A measure of 100 g of the AFB_1_ cultured maize was blended with 1 kg of diet/feed and then analyzed for AFB_1_ concentration and 58.19 mg/kg AFB1 concentration was recorded. Thereafter, this result was employed to calculate the amount of the cultured maize needed to be added to 1 kg of feed to achieve the desired 0.5 mg/kg AFB_1_, as follows:


$$\begin{array}{l} 100gAFB1cultured\;maize/1kgofbroiler\;diet=58.19mg/kgAFB1 \end{array}$$ (1)

$$\begin{array}{l} Xg\;cultured\;maize/1kg\;of\;broiler\;diet=0.5mg/kgAFB1 \end{array}$$ (2)

$$\begin{array}{l} X=\frac{100g\;x\;0.5\;mg/kg}{58.19\;mg/kg}=0.85g \end{array}$$

As a result, a total of 0.85 grams of the cultured maize was blended into 1 kg of the dietary treatments, namely AFTB, RCPA, and RCSA. Subsequent samples of these diets (AFTB, RCPA, and RCSA) were analyzed for AFB_1_ level, yielding 0.53 ± 0.01 mg/kg level.

### Experimental birds and welfare

Two hundred and forty-day-old broiler chicks of Cobb 500 breed were used for this study. The broiler day-old chicks were randomly distributed into four different experimental diet groups earlier stated and specified above, in a manner that each dietary group consists of 60 birds, each dietary group has six replicates and each replicate has 10 birds. In the first week of the feeding trial, the experimental pen’s temperature was maintained at 31 ± 2 °C. Subsequently, from weeks 2 to 3 of the study, the room temperature was gradually reduced by 2 °C per week. The broiler chickens were kept under the natural ambient temperature from weeks 4 to 6 of the rearing period. The lighting within the experimental room followed a diurnal pattern, encompassing 6 h of darkness during each 24-h cycle. This lighting regimen was upheld until 3 d before slaughtering.

### Performance characteristics, blood sample collection, and analysis

At the intervals of 7 d, critical growth metrics such as body weight, body weight gain, and feed consumption or intake, were meticulously documented. Additionally, the feed conversion ratio (FCR), which signifies the relationship between feed consumption and the increase in body weight was calculated as the fraction of the total feed intake/consumption and total weight gain. The mortality rate was also documented on daily basis as follows:


$$\begin{array}{lll} & Mortalityrate(\%)=\; & (Number\;of\;birds\;that\;died/Initial\;number\;of\;birds)*100 \end{array}$$

On day 42 of the feeding trial a total of 12 birds were selected from each experimental treatment (2 birds/replicate) were tagged adequately, restrained securely, and thereafter each bird’s neck was swabbed with methylated spirit and the sterile needle was inserted into the jugular vein on the side of the chicken’s neck, beneath the skin to draw blood into the syringe. Blood samples were divided into two types of sample bottles: one without any anticoagulant (plain bottles) and the other containing anticoagulant: ethylenediaminetetraacetic acid (EDTA). The samples in the plain bottles were subjected to spinning to separate the serum, which was then transferred to another set of plain bottles and stored at −20 °C for subsequent analysis. Hematological parameters, including red blood cell count (RBC), packed cell volume (PCV), hemoglobin concentration (HbC), mean cell hemoglobin concentration, mean cell volume, mean cell hemoglobin (MCH), white blood cell (WBC), granulocytes, lymphocytes, and monocytes were determined following the methodology outlined by Cheesbrough (2000). Furthermore, the total protein, globulin, albumin, alanine aminotransferase (ALT), aspartate aminotransferase (AST), creatinine, and cholesterol were determined with a Reflectron Plus 8C79 (Roche Diagnostic, GmbH Mannheim, Germany), were determined using commercial kits (Oloruntola et al., 2018). The serum catalase (Muhlisin et al., 2016), glutathione peroxidase (Cichoski et al., 2012), and 8-hydroxy-2’-deoxyguanosine (Zhang et al., 2013) were also determined.

### Statistical analysis

The data obtained were subjected to statistical analysis through SPSS software (version 20). A one-way analysis of variance (ANOVA) was utilized to assess the data. Subsequent to the ANOVA, the Duncan multiple range test, was employed to detect significant differences among the means of the distinct treatment groups. The statistical significance threshold was placed at *P* < 0.05.

## Results

Table 2 shows the outcomes of the performance evaluation for broiler chickens exposed to aflatoxin B_1_ and provided with diets containing red chili pericarp powder and seed powder supplements. The weight gain and feed intake of broiler chickens in the RCPA and RCSA groups were not significantly (*P* > 0.05) different from those in the CONT group. However, they were higher (*P* < 0.05) compared to the AFB_1_ group. The FCR for the RCPA and RCSA groups was comparable (*P* > 0.05) to CONT group. Additionally, the FCR for the AFTB group was similar (*P* > 0.05) to that of the RCPA group but significantly (*P* < 0.05) higher when compared to both the CONT and RCSA groups. The mortality rate of birds in the RCPA and RCSA groups did not differ significantly from the CONT group (*P* > 0.05) but was notably lower in comparison to those birds in AFTB group (*P* < 0.05).

**Table 2.** Performance of Aflatoxin B_1_ exposed broiler chickens fed red chili pericarp and seed powder supplemented diets

Parameters	CONT	AFTB	RCPA	RCSA	SEM	*P*-value
Initial weight, g/bird	40.19	40.21	40.23	40.21	0.01	0.84
Weight gain, g/bird	2,750.34^a^	2,252.77^b^	2,541.72^a^	2,712.67^a^	65.31	0.02
Feed consumption, g/bird	4,576.84^a^	4,197.68^b^	4,599.19^a^	4,576.20^a^	52.38	0.01
Feed conversion ratio	1.66^c^	1.86^a^	1.81^a^^,b^	1.69^b,c^	0.03	0.03
Mortality, %	1.11^b^	4.44^a^	1.67^b^	1.67^b^	0.42	0.01

Means within a row with different letters are significantly different (*P* < 0.05).

CONT, No aflatoxin B_1_ contamination; no supplementation.

AFTB, 0.5 mg/kg aflatoxin B_1_.

RCPA, 0.5 g/kg red chili fruit pericarp powder supplementation + 0.5mg/kg aflatoxin B_1_. RCSA, 0.5 g/kg red chili fruit seed powder supplementation + 0.5 mg/kg aflatoxin B_1_.

SEM, Standard error of the mean.

The results presented in Table 3 show the Hematological indices of aflatoxin B1 exposed broiler chickens fed red chili pericarp powder and red chili seed powder supplemented diets. The RBC count is significantly (*P* < 0.05) lower in the AFB_1_-exposed group (AFTB) compared to the control (CONT) group. However, the RCPA and RCSA groups have RBC counts similar (*P* > 0.05) to the CONT. The PCV is significantly (*P* < 0.05) reduced in birds in the AFTB group. Both the RCPA and RCSA groups’ birds show improved (*P* < 0.05) PCV values compared to the AFTB group, although they are still lower (*P* < 0.05) than those in the CONT group. Similar to RBC and PCV, the Hb concentration is significantly (*P* < 0.05) lower in the AFTB group. However, the RCPA and RCSA groups exhibit higher (*P* < 0.05) Hb concentration levels compared to the AFTB group. The total WBC count is significantly (*P* < 0.05) reduced in the AFTB group. The RCPA and RCSA treatment groups do not show significant (*P* > 0.05) differences from the CONT group in terms of WBC count. AFB_1_ exposure results in a significant (*P* < 0.05) decrease in granulocyte count, and this reduction is partially mitigated by Red Chili pericarp powder supplementation. AFB_1_ exposure leads to a significant (*P* < 0.05) reduction in lymphocyte count. However, the RCPA and RCSA treatment groups exhibit higher lymphocyte counts compared to the AFTB group.

**Table 3.** Hematological indices of aflatoxin B_1_ exposed broiler chickens fed red chili pericarp and seed powder supplemented diets

Parameters	CONT	AFTB	RCPA	RCSA	SEM	*P*-value
Red blood cells, ×10^12^/l	3.25^a^	2.35^b^	3.00^a^	3.15^a^	0.11	0.01
Packed cell volume, %	37.50^a^	30.00^c^	33.50 ^b^	36.50^a^	0.96	0.01
Hemoglobin conc., g/dl	12.50^a^	10.00^c^	11.16^b^	12.17^a^	0.32	0.02
Mean cell hemoglobin conc., g/dl	33.33	33.34	33.35	33.33	0.00	0.33
Mean cell volume, fl	115.35	128.94	111.56	116.09	3.17	0.24
Mean cell hemoglobin, pg	38.45	42.98	37.18	36.69	1.05	0.25
White blood cells, ×10^9^/l	3.50^a^	2.80^c^	3.20^ab^	3.40^a^	0.11	0.04
Granulocytes, ×10^9^/l	1.02^a^	0.91^ab^	0.60^b^	1.19^a^	0.08	0.03
Lymphocytes, ×10^9^/l	2.07^a^	1.19^b^	1.98^a^	2.12^a^	0.12	0.01
Monocytes, ×10^9^/l	0.41	0.69	0.62	0.08	1.00	0.12

Means within a row with different letters are significantly different (*P* < 0.05).

CONT, No aflatoxin B_1_ contamination; no supplementation.

AFTB, 0.5 mg/kg aflatoxin B_1_.

RCPA, 0.5 g/kg red chili fruit pericarp powder supplementation + 0.5mg/kg aflatoxin B_1_.

RCSA, 0.5 g/kg red chili fruit seed powder supplementation + 0.5 mg/kg aflatoxin B_1_.

SEM, Standard error of the mean.

Table 4 reveals the serum proteins and enzymes of aflatoxin B_1_ exposed broiler chickens fed red chili pericarp powder and red chili seed powder supplemented diets. The concentrations of serum total protein and globulin in both the RCPA and RCSA groups were not significantly different (*P* > 0.05) from those in the control (CONT) group. However, they were notably higher (*P* < 0.05) in comparison to those in the CONT group. The albumin concentrations of birds in the RCPA and RCSA treatment groups did not show a significant difference (*P* > 0.05) compared to those in the CONT group and were also similar (*P* > 0.05) to those in the AFTB group. The alanine aminotransferase concentrations of birds in the RCPA and RCSA groups were comparable (*P* > 0.05) to the broiler chickens in the CONT group but significantly lower (*P* < 0.05) than the broiler chickens in the AFTB group. As for aspartate aminotransferase concentrations, the RCPA and RCSA groups exhibited no significant difference (*P* > 0.05) compared to those in the CONT group, but they were significantly lower (*P* < 0.05) than those in the AFTB group. The creatinine levels did not display a significant difference (*P* = 0.95) across all the treatment groups. Serum cholesterol levels of birds in the AFTB, RCPA, and RCSA treatment groups were not significantly different (*P* > 0.05) from each other, but they were significantly lower (*P* < 0.05) than those in the CONT treatment groups.

**Table 4.** Serum proteins and enzymes of aflatoxin B_1_ exposed broiler chickens fed red chili pericarp and seed powder supplemented diets

Parameters	CONT	AFTB	RCPA	RCSA	SEM	*P*-value
Total protein, g/l	51.36^a^	42.53^b^	48.74^a^	50.16^a^	1.18	0.01
Globulin, g/l	34.99^a^	28.42^b^	33.05^a^	34.94^a^	0.96	0.02
Albumin, g/d	16.36^a^	14.11^b^	15.69^ab^	15.21^ab^	0.34	0.12
Alanine aminotransferase, IU/L	58.74^bc^	82.20^a^	63.81^b^	48.59^c^	4.33	0.02
Aspartate aminotransferase, IU/L	100.99^b^	124.82^a^	106.96^b^	101.84^b^	3.07	0.01
Creatinine, µmol/L	37.27	43.43	39.01	39.58	3.52	0.95
Cholesterol, mmol/L	3.07^a^	2.98^b^	2.82^b^	2.30^b^	0.11	0.02

Means within a row with different letters are significantly different (*P* < 0.05).

CONT, No aflatoxin B_1_ contamination; no supplementation.

AFTB, 0.5 mg/kg aflatoxin B_1_.

RCPA, 0.5 g/kg red chili fruit pericarp powder supplementation + 0.5mg/kg aflatoxin B_1_.

RCSA, 0.5 g/kg red chili fruit seed powder supplementation + 0.5 mg/kg aflatoxin B_1_.

SEM, Standard error of the mean.

Table 5 displays the serum levels of oxidative stress enzymes and the deoxyribonucleic acid destroyer biomarker in broiler chickens exposed to aflatoxin B_1_ and fed diets supplemented with red chili pericarp powder and red chili seed powder. The serum catalase levels of birds in the RCPA and RCSA groups did not significantly differ (*P* > 0.05) from those in the CONT group. In contrast, the serum catalase levels in the AFTB group were similar to those in the RCSA group but notably lower (*P* < 0.05) than those in the CONT and RCSA groups. The serum peroxidase levels of broiler chickens in the AFTB group were notably lower (*P* < 0.05) than those observed in the CONT, RCPA, and RCSA groups. Additionally, the serum glutathione levels of the broiler chickens in the RCPA group were found to be similar (*P* > 0.05) to those of the CONT group.

**Table 5.** Serum oxidative stress enzymes and deoxyribonucleic acid destroyer biomarker of aflatoxin B1 exposed broiler chickens fed red chili pericarp and seed powder supplemented diets

Parameters	CONT	AFTB	RCPA	RCSA	SEM	*P*-value
CAT, ng/mgprotein	75.81^a^	58.17^b^	73.05^a^	67.92^ab^	2.65	0.05
GPx, ml/mgprotein	178.38^a^	125.92^c^	167.61^ab^	152.89^b^	6.56	0.01
OHdG, gn/ml	148.62^c^	197.19^a^	153.41^bc^	184.12^ab^	7.42	0.02

Means within a row with different letters are significantly different (*P* < 0.05).

CONT, No aflatoxin B_1_ contamination; no supplementation.

AFTB, 0.5 mg/kg aflatoxin B_1_.

RCPA, 0.5 g/kg Red Chili fruit pericarp powder supplementation + 0.5mg/kg aflatoxin B_1_.

RCSA, 0.5 g/kg red chili fruit seed powder supplementation + 0.5 mg/kg aflatoxin B_1_.

SEM, standard error of the mean; CAT, catalase; GPx, glutathione peroxidase; OHdG, 8-hydroxy-2’-deoxyguanosine.

The serum levels of 8-hydroxy-2’-deoxyguanosine of broiler chickens in the AFTB group were higher (P < 0.05) than those observed in the CONT and RCPA treatment groups. The 8-hydroxy-2’-deoxyguanosine levels of birds in the RCSA and RCPA groups were comparable (*P* > 0.05) to those in CONT.

## Discussion

Red chili is known for its nutraceutical properties, which include a rich content of bioactive compounds such as capsaicinoids, carotenoids, and flavonoids. These compounds have been reported to possess antioxidant, anti-inflammatory, and immune-enhancing properties (Villa-Rivera and Ochoa-Alejo, 2020; Oloruntola et al., 2023). The dietary supplementation of red chili pericarp and seed powder in the diets of broiler chickens may have contributed to the observed positive effects on weight gain, feed intake, and FCR (Abd El Hack et al., 2022). The antioxidants present in red chili may have played a role in mitigating the oxidative stress induced by AFB_1_ (Oloruntola et al., 2023). For instance, AFB_1_ is known to induce the production of free radicals within the body, leading to oxidative stress (Jobe et al., 2023), and antioxidants, such as those found in red chili (e.g., capsaicinoids, vitamin C, carotenoids, flavonoids, and phenolic compounds), can scavenge these free radicals and prevent them from causing damage to cells and tissues (Azlan et al., 2022). Aflatoxin B_1_ is a well-known mycotoxin known to be highly toxic and can deteriorate the health and performance of poultry, causing reduced growth, immunosuppression, and an increased mortality rate (Yunus et al., 2011). The results in this study are consistent with the common negative impact of AFB_1_ on broiler chickens when not counteracted by interventions (Tavangar et al., 2021).

In the study, the weight gain and feed intake of broiler chickens in the RCPA and RCSA groups were similar to the uncontaminated control (CONT) group, indicating that the red chili supplementation was effective in maintaining growth performance in the presence of AFB_1_ contamination (Tavangar et al., 2021). Some components in red chili, such as capsaicin, have anti-inflammatory properties (Shang et al., 2017; Oloruntola et al., 2023), and inflammation is a common response to cellular damage (Rock and Kono, 2008). Knowing that AFB_1_ exposure can trigger inflammation, red chili supplementation may also help to reduce the inflammatory response and prevent the associated negative impact on growth, as observed in this study. The improved FCR in these groups further underscores the potential of red chili to enhance feed efficiency even in challenging conditions. Specifically, capsaicin, one of the active compounds in red chili, can stimulate digestive processes (Li et al., 2022a), may increase the production of digestive enzymes and improve nutrient absorption in the gastrointestinal tract and consequently help chickens extract more nutrients from their feed, even when it contains AFB_1_ (Li et al., 2022a; Herrero-Encinas et al., 2023). The lower mortality rate observed in the RCPA and RCSA groups compared to the AFB_1_ group is an important finding because it suggests that red chili supplementation may offer a degree of protection against the pathogenic effects of AFB_1_, possibly through its antioxidant and immune-boosting properties and activities (Hatipoglu and Keskin, 2022). AFB_1_ is known to suppress the immune system, making chickens more vulnerable to infections and diseases. Therefore immune-boosting compounds such as flavonoids and carotenoids in phytogenic supplements (i.e., red chili pericarp powder and red chili seed powder) used in this study could have strengthened the chickens’ immune response of the birds (Philip et al., 2023), enabling them to defend against pathogens more effectively and consequently reduces the risk of mortality due to aflatoxicosis (Li et al., 2022b).

The significant decrease in RBC count in the AFTB (AFB_1_-exposed group) is consistent with the well-documented adverse effects of AFB_1_ on hematological parameters (Mahfouz and Sherif, 2015). AFB_1_ can induce bone marrow damage and impair erythropoiesis, leading to decreased RBC production (Zivot et al., 2018). In contrast, both the RCPA and RCSA groups showing RBC counts similar to the control group suggests that red chili supplementation, either in the form of pericarp powder or seed powder, may offer protection against AFB1-induced RBC depletion (Cervantes-Hernández et al., 2019). The antioxidants and bioactive compounds in red chili may help counteract the mycotoxin’s impact on RBC production (Al Balushi et al., 2019). The significant reduction in PCV in the AFTB (AFB_1_-exposed group) reflects the decreased proportion of RBCs in the blood, a hallmark of AFB1-induced anemia (Gholami-Ahangaran and Zia-Jahromi, 2012). AFB_1_ is primarily metabolized in the liver, and the liver plays a crucial role in synthesizing and regulating the production of various blood components, including RBCs (Liu et al., 2020). AFB_1_ can cause damage to the liver, impairing its capability to support erythropoiesis. This decrease in RBC count results in a reduced PCV, as PCV measures the proportion of blood volume occupied by RBCs. AFB_1_ can disrupt hemoglobin synthesis and erythropoiesis, contributing to reduced PCV (Khandros and Weiss, 2010). The RCPA and RCSA groups exhibited improved PCV values compared to the AFTB group, although not fully restored to the control level. This suggests that red chili supplementation may help mitigate the anemia induced by AFB1, possibly by reducing oxidative stress and inflammation (Cotoraci et al., 2021). The significant reduction in Hb concentration in the AFB_1_-exposed group is in line with the mycotoxin’s known impact on hemoglobin synthesis and erythropoiesis (Lei et al., 2021). In contrast, both the RCPA and RCSA groups showed higher Hb concentration levels compared to the AFTB treatment group, suggesting that the red chili supplementation could help to maintain or restore the normal Hb concentration levels in the presence of AFB_1_ contamination. The antioxidants and bioactive compounds in red chili may play a role in this protective effect (Cotoraci et al., 2021; Bal et al., 2022). The reduced total WBC count of birds in the AFTB group is indicative of AFB_1_-induced immunosuppression, which can make chickens more susceptible to infections (Ochieng et al., 2021). The RCPA and RCSA groups did not show significant differences in WBC counts compared to the control group. This suggests that red chili supplementation may help maintain the total WBC count and potentially support immune function in AFB_1_-exposed broiler chickens. Red chili supplements, being natural antioxidant phytogen could potentially support immune function in AFB_1_-exposed broiler chickens by reducing inflammation, enhancing the immune response, neutralizing oxidative stress, and protecting WBCs (Li et al., 2022b). The significant reduction in granulocyte and lymphocyte count in the AFTB group indicates incidence of immunosuppression and an increased risk of infections (Sabourin et al, 2006). The RCSA groups exhibited improved granulocyte counts and the RCPA and RCSA groups exhibited improved lymphocyte counts, compared to the AFB_1_ group, suggesting that red chili pericarp powder and red chili seed powder supplementation may enhance the immune response and mitigate the immunosuppressive effects of AFB_1_ (Rasouli et al., 2022).

Both the RCPA and RCSA groups exhibited similar total protein and globulin concentrations to the control (CONT) group, and these values were significantly higher compared to the CONT group. This suggests that red chili supplementations had a positive effect on the maintenance of total protein and globulin levels, which play a crucial role in immune function (Reda et al., 2020). The observed stability in these serum proteins indicates that red chili pericarp powder and seed powder supplementation may counteract the AFB_1_-induced reduction in protein levels possibly by providing antioxidant protection, supporting the immune system, reducing inflammation, and safeguarding the liver (Villa-Rivera and Ochoa-Alejo, 2020; Rasouli et al., 2022). These effects may contribute to maintaining or restoring protein levels in the serum of broiler chickens exposed to AFB_1_. The albumin concentrations in the RCPA and RCSA groups being similar to those in the CONT group and simultaneously, also similar to the AFB_1_ group in this study suggests that red chili supplementation did not have a significant impact on albumin levels. This may be due to the complexity of albumin regulation, individual variability, and the specific conditions of the study (Bern et al., 2015). While red chili supplementation may have beneficial effects on other parameters, its influence on albumin levels might be limited due to the interplay of factors, such as the duration of supplementation, dosage, and individual variability in response to dietary supplement under study.

The RCPA and RCSA groups exhibited ALT and AST concentrations that were comparable to the CONT group, but significantly lower than those in the AFTB group. This indicates that red chili supplementation was effective in reducing the elevated levels of ALT and AST induced by AFB_1_. Elevated ALT and AST are markers of liver damage, and the mitigation of these enzymes suggests a protective effect of red chili against AFB_1_-induced liver injury. Phytogen or nutraceutic feed supplements such as red chili pepper, and particularly its active compound capsaicin, may protect the liver from AFB1-induced damage by providing antioxidant support, reducing inflammation, enhancing detoxification, improving blood flow, preventing fibrosis, and modulating the immune response (Cheng et al., 2023). Serum cholesterol levels in the AFTB, RCPA, and RCSA groups were similar to each other, but they were significantly lower compared to the CONT group. Aflatoxin B_1_ (AFB_1_) exposure is known to disrupt normal lipid metabolism and can lead to dyslipidemia, which is an abnormal level of lipids (including cholesterol) in the blood (James et al., 2022). Red chili, particularly capsaicin, has been studied for its potential lipid-lowering effects. It may influence the metabolism of lipids, including cholesterol, by enhancing lipid breakdown and reducing lipid synthesis in the liver (Gong et al., 2022). Therefore, the similarity in serum cholesterol levels among the AFTB, RCPA, and RCSA groups, while significantly lower compared to the CONT group, may be due to a combination of AFB_1_-induced dyslipidemia and the potential lipid-lowering effects of Red Chili supplementation. This suggests that Red Chili might help partially to mitigate the AFB_1_-induced disruption of lipid metabolism, but complete restoration of cholesterol levels may not be achieved under the experimental conditions.

An increase in serum catalase, an antioxidant enzyme, typically indicates that the body is responding to higher levels of oxidative stress or the presence of excess ROS (Bhattacharyya et al., 2014). This increase is an adaptive response aimed at neutralizing the harmful effects of oxidative stress. In the RCPA and RCSA groups (red chili pericarp and seed supplementation), the serum catalase levels were not significantly different from those in the control (CONT) group, suggesting that red chili supplementation did play roles in stabilizing the oxidative status of the aflatoxin B_1_ exposed birds. The roles of phytogens or phytochemicals in stabilizing the oxidative status of animals were reported (Lee et al., 2017). In addition, AFB_1_ can cause a reduction in serum catalase by promoting oxidative stress, depleting antioxidant defenses, directly affecting enzyme activity, triggering inflammation, causing cellular and tissue damage, and overall impacting the body’s health and ability to maintain normal antioxidant enzyme levels (Benkerroum, 2020b). Therefore, the catalase level of birds in the AFB_1_-exposed group, being similar to those in the RCSA group but notably lower than those in both the CONT and RCPA groups depicts the incidence of oxidative damage caused by AFB_1_ and that red chili seed powder was able to reduce the negative effects of aflatoxin B1 on the oxidative status of the birds to a limited extent.

The observed significant reduction in serum peroxidase levels in the AFB_1_-exposed group compared to the CONT, RCPA, and RCSA groups highlights the adverse effects of AFB_1_ on the antioxidant defense system (Sarker et al., 2023). Peroxidases are key antioxidant enzymes that play a critical role in neutralizing ROS and protecting cells from oxidative damage (Bhattacharyya et al., 2014). A decrease in peroxidase activity suggests that AFB_1_ induces oxidative stress by promoting the production of ROS (Dai et al., 2022). This finding is consistent with previous studies that have reported the oxidative stress-inducing properties of AFB_1_ (Tao et al., 2021).

The 8-hydroxy-2’-deoxyguanosine (8-OHdG) is a specific type of DNA lesion that indicates the presence of oxidative damage to DNA (AbuArrah et al., 2021). The significantly higher 8-OHdG levels in the AFB_1_ group compared to the CONT and RCPA groups indicate that AFB_1_ exposure led to increased oxidative DNA damage. This finding aligns with the known ability of AFB_1_ to induce oxidative stress and DNA damage (Yilmaz et al., 2017). The similarity in 8-OHdG levels between the RCSA and RCPA groups (*P* > 0.05) suggests that both red chili supplementation strategies (fruit pericarp and seed powder) had a comparable impact on reducing oxidative DNA damage in the presence of AFB_1_. Red chili is known to contain various antioxidants, including capsaicin, ascorbic acid, and carotenoids, which can neutralize free radicals and reduce oxidative stress (Bal et al., 2022). These antioxidants have the ability to scavenge ROS generated during oxidative stress, thus protecting DNA from damage (Bal et al., 2022; Sanatomi, 2023).

## Conclusion

Conclusively, 0.5 g/kg supplementation with red chili pericarp powder (RCPA) and Red Chili seed extract (RCSA) demonstrated positive effects on AFB_1_ exposed broiler chickens’ performance characteristics: body weight gain, feed consumption, FCR, and mortality rate, comparable to the control group. The hematological parameters such as RBC count, PCV, and Hb concentration also showed improvements in the birds in the RCPA and RCSA groups compared to those in the AFTB group. Furthermore, these supplements (0.5 g/kg red chili pericarp powder and 0.5 g/kg red chili seed powder) exhibited protective effects on liver enzymes and antioxidant status. These findings suggest the potential of 0.5 g/kg red chili pericarp powder and 0.5 g/kg red chili seed powder in mitigating the adverse effects of AFB_1_ exposure in broiler chickens.
